# Prostate-Specific Membrane Antigen Positron Emission Tomography-Detected Intrahepatic Cholangiocarcinoma in a Patient With Metastatic Castration-Resistant Prostate Cancer: Dual Cancer Management With Pluvicto and Intensity-Modulated Radiation Therapy

**DOI:** 10.7759/cureus.90145

**Published:** 2025-08-15

**Authors:** Harshad R Kulkarni, Kevin A Maupin, Emerson Lim, Eric P Buth, Brandon R Mancini

**Affiliations:** 1 Theranostics, BAMF Health, Grand Rapids, USA; 2 Radiology, Michigan State University College of Human Medicine, Grand Rapids, USA; 3 Oncology, Corewell Health, Grand Rapids, USA; 4 Radiation Oncology, Corewell Health, Grand Rapids, USA

**Keywords:** intensity-modulated radiation therapy (imrt), intrahepatic cholangiocarcinoma (icc), prostate cancer, psma imaging and therapy, spect and pet imaging

## Abstract

Intrahepatic cholangiocarcinoma (ICC) is a rare hepatobiliary malignancy often diagnosed at advanced stages due to its silent progression. Prostate-specific membrane antigen (PSMA) PET/CT, primarily used in prostate cancer, can incidentally detect unrelated malignancies. In a patient with metastatic castration-resistant prostate cancer (mCRPC), PSMA PET/CT revealed a liver lesion initially presumed metastatic. However, lack of uptake on post-therapy single-photon emission computed tomography/CT (SPECT/CT) prompted further evaluation, and MRI with biopsy confirmed ICC. The patient received intensity-modulated radiation therapy (IMRT) for ICC, stereotactic body radiation therapy (SBRT) for prostate metastases, and continued Lu-177 PSMA-617 therapy. This case highlights the incidental diagnostic value of PSMA PET and the importance of post-therapy imaging and multidisciplinary care in managing complex cancer presentations.

## Introduction

Prostate-specific membrane antigen (PSMA) positron emission tomography/computed tomography (PET/CT) has revolutionized the management of metastatic prostate cancer, significantly improving the detection of small-volume disease and guiding targeted therapies, such as Lu-177 PSMA-617 (LuPSMA) [1] for metastatic castration-resistant prostate cancer (mCRPC). However, non-physiological PSMA expression is not exclusive to prostate cancer and has been observed in a variety of malignancies, including intrahepatic cholangiocarcinoma (ICC) and hepatocellular carcinoma, where PSMA expression is often observed in tumor neovasculature [2,3]. Although PSMA is expressed on the endothelium of these tumors, allowing for theoretical targeting and treatment with LuPSMA, this endothelial expression results in minimal retention of PSMA-targeted molecules within the tumor. Consequently, these tumors receive very low radiation doses compared to prostate cancer, where PSMA is expressed directly by the cancer cells [4]. The high tumor radiation dose in prostate cancer metastases results from the efficient internalization and prolonged intracellular retention of therapeutic radioligands such as LuPSMA. While rare, prostate cancer patients can also have synchronous primary liver tumors that are indistinguishable by PSMA PET. However, due to the poor long-term retention of PSMA-targeting radiopharmaceuticals, these non-prostate tumors can be readily discerned by poor retention of LuPSMA in 24-hour post-therapeutic single-photon emission computed tomography/CT (SPECT/CT) imaging [4].

ICC is a rare hepatobiliary malignancy that is often diagnosed in advanced stages due to its asymptomatic progression. This case illustrates an incidentally detected intrahepatic ICC on PSMA PET/CT in a patient undergoing LuPSMA treatment for mCRPC and highlights a personalized dual-cancer management approach.

## Case presentation

This retrospective case report analyzes a patient with mCRPC who was treated with LuPSMA between July 2023 and September 2024 at BAMF Health following confirmation of PSMA-expressing disease on PSMA PET imaging. Given the evolving disease course, follow-up PSMA PET/CT was performed before the third cycle and after the sixth cycle of LuPSMA at BAMF Health, Grand Rapids, USA. While the retrospective analysis was conducted at a single institution (BAMF Health), the authors had additional affiliations, with two contributing authors, a medical oncologist and radiation oncologist, respectively, primarily affiliated at another institution (Corewell Health), but were involved in patient care as well as scientific collaboration.

Patient history and prior treatment

A 69-year-old male was diagnosed with stage IIIC (pT3a pN0 cM0), Gleason 4+5=9 (Grade Group 5) prostatic adenocarcinoma, with an initial prostate-specific antigen (PSA) level of 8.66 ng/mL. His treatment course included radical prostatectomy with pelvic lymph node dissection in March 2021, followed by adjuvant external beam radiation therapy (EBRT) in July 2021. Despite initial disease control, he experienced biochemical recurrence in January 2022, prompting the initiation of docetaxel chemotherapy in February 2022. Due to further progression, the patient was transitioned to abiraterone in May 2022 and subsequently to enzalutamide in November 2022.

PSMA PET/CT imaging

A baseline PSMA PET/CT scan was performed at another institution using a conventional PET/CT scanner in May 2023. A vertex to thigh PET/CT was performed (three minutes/bed position, eight bed positions) 75 minutes after the injection of 9 mCi (333 MBq) of Pylarify (fluorine-18 (F-18) piflufolastat). The disease restaging revealed PSMA-positive pelvic lymph nodes and a progressive PSMA-avid hepatic lesion, raising suspicion for metastatic involvement. Based on the scan findings in mCRPC following treatment with an androgen receptor pathway inhibitor and taxane-based chemotherapy, the patient was deemed a suitable candidate for PSMA-targeted radioligand therapy with LuPSMA. For follow-up PSMA PET/CT imaging, the patient received an injection of 111-259 MBq (3-7 mCi) of Illuccix (gallium-68 (Ga-68) gozetotide or Ga-68 PSMA-11) and underwent imaging 50-75 minutes after injection using a long-axis-field-of-view PET/CT scanner. The PET acquisition time was three minutes.

PSMA-targeted radioligand therapy (PRLT), SPECT/CT imaging, and diagnosis of ICC

The patient received approximately 200 mCi of LuPSMA per cycle, following the prescribing guidelines. The actual administered dosage ranged from 188.3 to 205.6 mCi per cycle, with no dosage reductions required. The number of treatment cycles was determined based on PSA kinetics, molecular tumor burden response via post-therapeutic single-photon emission computed tomography (SPECT/CT) imaging, and comprehensive evaluations of hematologic and chemistry parameters, as well as overall clinical status. Whole-body SPECT/CT imaging was performed 20-24 hours after infusion to assess LuPSMA biodistribution, tumor uptake, and volume. Both the 113 keV and 208 keV energy peaks of Lu-177 were acquired for quantitative analysis. Given the complex disease presentation, imaging findings were integrated with MRI and histopathological results to guide treatment adjustments, particularly in response to the incidentally detected intrahepatic cholangiocarcinoma (ICC).

**Figure 1:**
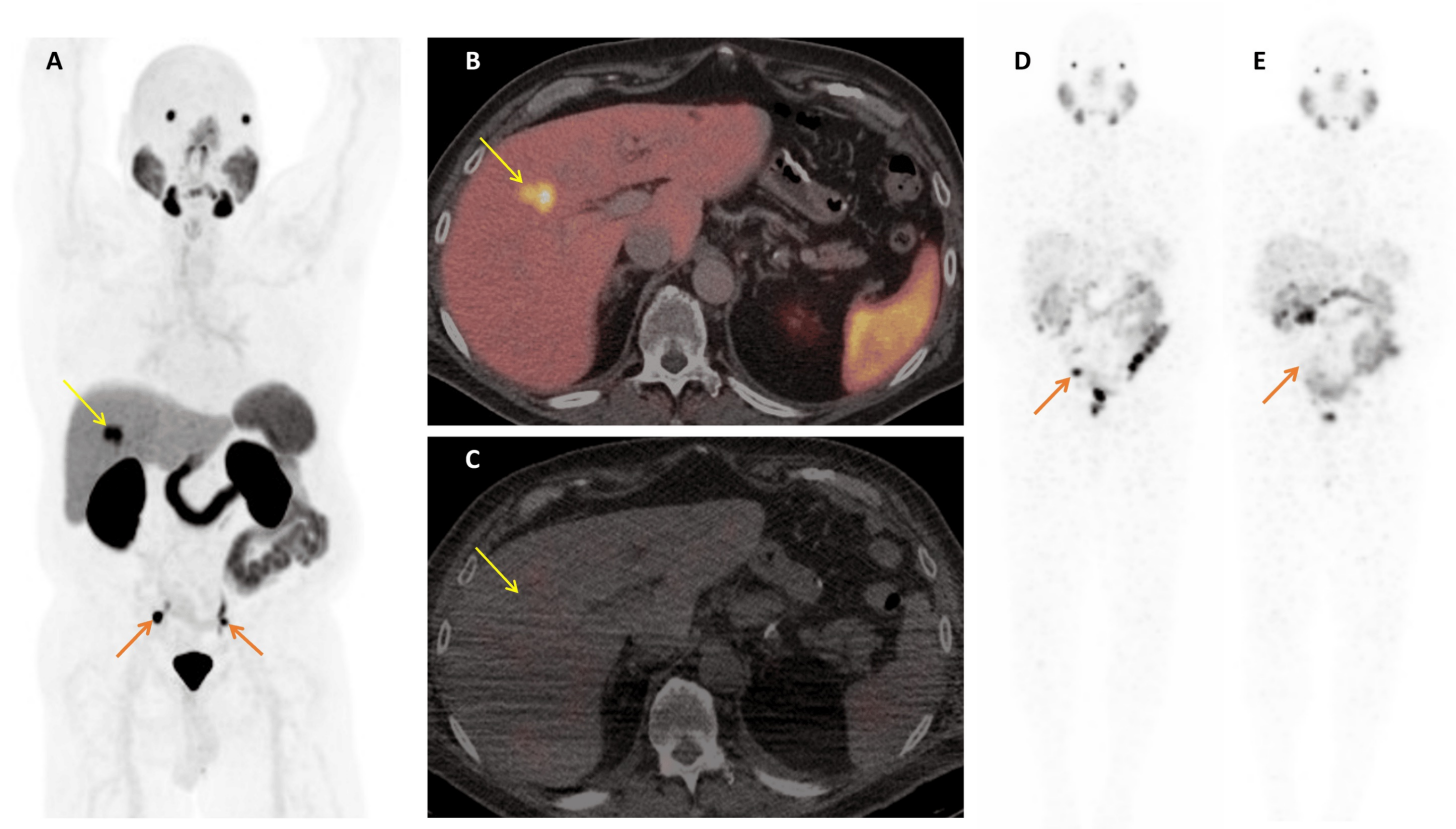
Initiation of prostate-specific membrane antigen (PSMA)-targeted radioligand therapy Pylarify (F-18 DCFPyL, a PSMA-targeted PET ligand) PET/CT showed a PSMA-positive liver lesion in the right hepatic lobe (SUVmax 14.6, yellow arrow) and PSMA-expressing right and left iliac lymph node metastases (orange arrows). Compared to prior imaging (not shown), the liver lesion had increased in size. After the first cycle of LuPSMA therapy, SPECT/CT demonstrated no significant long-term retention of LuPSMA in the liver lesion (yellow arrow), but persistent uptake was noted in the right iliac lymph node (orange arrow). Following the second therapy cycle, the right iliac node showed a marked decrease in PSMA expression and size on SPECT/CT (orange arrow).

**Figure 2:**
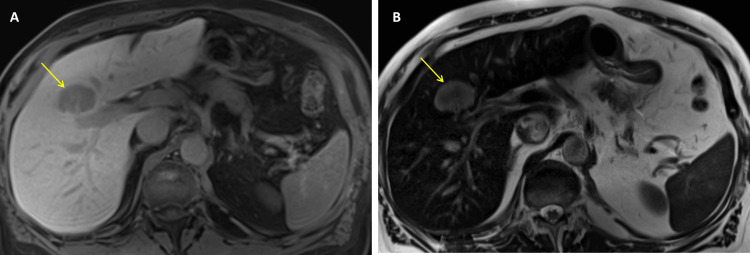
MRI and diagnosis of intrahepatic cholangiocarcinoma Magnetic resonance imaging (MRI – A, axial T1; B, axial T2) after the initial LuPSMA cycle showed an interval increase in size of an isolated right hepatic lobe mass (yellow arrow), now measuring 3.1 × 4.1 cm (previously 1.3 cm on prior MRI, not shown), compatible with neoplasm such as hepatocellular carcinoma or possible metastasis. No other suspicious hepatic lesions were identified.

Following the first cycle of LuPSMA therapy in July 2023, a post-treatment SPECT/CT scan demonstrated radioligand uptake in pelvic lymph nodes but no uptake within the liver lesion, raising suspicion for a non-prostate malignancy (Figure 1). Given the atypical uptake pattern in the liver, further evaluation was warranted. Subsequent MRI of the abdomen identified a lesion atypical for prostate cancer metastasis (Figure 2). A CT-guided liver biopsy confirmed an ICC. The pathology revealed sheets of moderately pleomorphic cells with abundant eosinophilic cytoplasm and scattered mitoses, along with focal necrosis. The clinical stage (American Joint Committee on Cancer (AJCC), 8th Edition) of the 4.1 cm measuring ICC in the right lobe segment 4 (S4) of the liver was IA (cT1a cN0 cM0).

The patient's disease course was characterized by significant fluctuations in PSA levels (Table 1). Following the first cycle of LuPSMA therapy in July 2023, a marked decrease in PSA was observed, correlating with a significant reduction in the size and PSMA uptake of the iliac lymph node metastases on the SPECT/CT imaging after the second LuPSMA cycle in August 2023 (Figure 2).

**Table 1:** Timeline of treatments received and prostate-specific antigen (PSA) course before and after Lu-177 PSMA-617 (LuPSMA) therapy The table details the chronological progression of PSA levels alongside key treatment events, highlighting fluctuations in response to therapy. LuPSMA treatment cycles are bolded for reference.

Weeks from Baseline	PSA (ng/mL)	Event
0	4.77	PSA Measurement
1w	-	Cycle #1 of Pluvicto
3w	5.19	PSA Measurement
6w	3.49	PSA Measurement
7w	-	Cycle #2 of Pluvicto
9w	2.92	PSA Measurement
10w	-	Liver Biopsy (Confirmed ICC)
12w	2.73	PSA Measurement
21w	-	Completion of IMRT to ICC & SBRT to lymph node metastases
37w	75.70	PSA Measurement
43w	-	Cycle #3 of Pluvicto
45w	153.00	PSA Measurement
48w	98.50	PSA Measurement
49w	-	Cycle #4 of Pluvicto
51w	66.30	PSA Measurement
53w	-	Thyroidectomy (Hyperplastic Nodule)
54w	57.70	PSA Measurement
55w	-	Cycle #5 of Pluvicto
57w	74.30	PSA Measurement
60w	61.00	PSA Measurement
61w	-	Cycle #6 of Pluvicto
63w	57.90	PSA Measurement
66w	42.20	PSA Measurement
70w	57.50	PSA Measurement
74w	80.70	PSA Measurement
79w	-	Completion of IMRT to Retroperitoneal Lymph Node Metastases
81w	33.60	PSA Measurement

Therapy of ICC and SBRT of pelvic lymph node metastases

Due to the patient's preference to avoid a partial hepatectomy, the ICC was treated with external beam radiation (EBRT) in the form of an intensity-modulated radiotherapy (IMRT). Additionally, as he planned to be away from the treating center for the winter, it was decided to defer the systemic therapy of mCRPC with LuPSMA and favor definitive management of the pelvic lymph nodes with stereotactic body radiotherapy (SBRT). IMRT (4500 cGy in 15 fractions) was administered for ICC in S4 of the liver in addition to SBRT to the bilateral pelvic lymph nodes (3500 cGy in five fractions).

Re-introduction of LuPSMA and therapy response assessment

**Figure 3:**
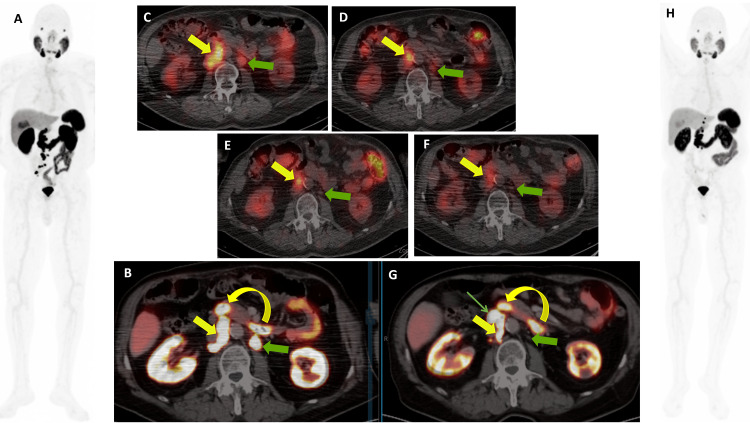
Follow-up prostate-specific membrane antigen (PSMA) PET and single-photon emission computed tomography (SPECT) imaging Follow-up Illuccix (Ga‑68 PSMA‑11) PET/CT (A, B) after two cycles of LuPSMA, IMRT to the ICC, and SBRT to pelvic nodes showed new retroperitoneal (solid yellow arrows) and pelvic lymph node metastases (the curved yellow arrow represents physiological intestinal uptake). SPECT/CT after the third, fouth, fifth, and sixth cycles (C–F) showed marked response, with decreased size and PSMA uptake in retroperitoneal nodes (solid yellow arrows) and complete regression of the left para‑aortic node (solid green arrow). PET/CT 10 weeks after the sixth cycle (G, H) showed mixed response: regression of many lymph nodes but new metastases in the precaval (outlined green arrow) and upper retroperitoneal regions. Decreased PSMA uptake in the ICC (A, H – MIP images) was also seen.

The patient returned to our center in the Spring 2024, and LuPSMA therapy was reintroduced in April 2024 due to mCRPC progression. Restarting treatment led to another period of PSA reduction and decreased tumor burden (Figure 3). The sixth cycle of LuPSMA was completed in September 2024. The patient tolerated LuPSMA without any side effects, maintaining a good quality of life. Blood analysis demonstrated no evidence of significant hematological, renal, or hepatic toxicity, except for mild anemia with leukocytopenia. The follow-up PSMA PET/CT in November 2024 revealed a mixed response with regression in the existing lymph node metastases but newly developed lesions in the retroperitoneal lymph node chain (Figure 3).

**Figure 4:**
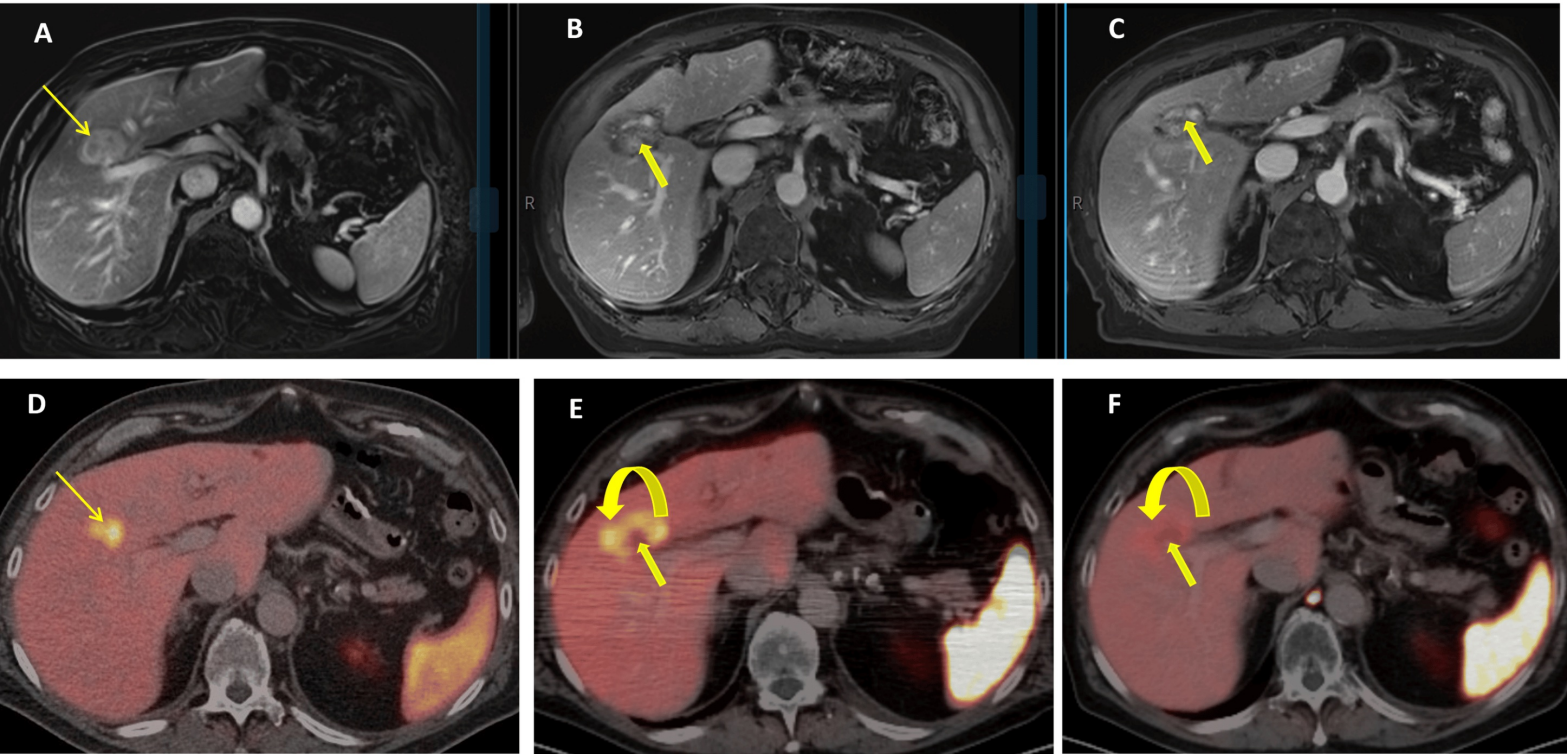
Follow-up MRI The course of ICC is shown on axial T1 MRI (A–C) and serial PSMA PET/CT (D–F). On MRI, the first post-IMRT scan (B) demonstrates central necrosis (bold arrow) with a surrounding hypoperfused zone, consistent with post-procedure inflammation. A later follow-up (C) shows reduction of the ablation zone and no residual disease. On PSMA PET/CT, the initially PSMA-avid ICC (D) develops central necrosis (bold arrow) and a surrounding area of PSMA overexpression (curved arrow) five months after IMRT (E), reflecting inflammation or neovasculature, in concordance with MRI (B). At 12 months post IMRT (F), the ICC remains regressed (bold arrow), with resolution of the surrounding PSMA uptake.

Repeat MRI after IMRT demonstrated a decrease in ICC size and treatment response, which correlated with PSMA PET findings (Figure 4).

## Discussion

This case underscores the incidental detection of a second malignancy (ICC) via PSMA PET/CT in a patient undergoing imaging for mCRPC. The findings highlight the importance of a multimodal approach integrating molecular imaging, theranostics, and targeted radiotherapy in the management of patients with complex oncologic presentations such as multiple malignancies.

PSMA is strongly overexpressed in prostate cancer, with higher levels associated with advanced disease and metastatic progression [5]. Resting endothelial cells show little-to-no PSMA expression. However, PSMA has been shown to be upregulated in the often torturous neovasculature of most solid tumors, including ICC [6]. The detection of PSMA-avid lesions outside the prostate in oncologic imaging, as in this case, necessitates careful interpretation, particularly when lesions exhibit atypical uptake patterns.

Liver tumors, particularly cholangiocarcinomas, are often highly vascular, making it unsurprising that PSMA overexpression is found in these tumors. Chen et al. [2] analyzed PSMA expression in 446 formalin-fixed, paraffin-embedded liver tumors, including 213 HCC, 203 ICCs, and 30 cases of liver cirrhosis. They found PSMA expression in 86.8% of HCCs and 79.3% of ICCs, but only 6.6% of liver cirrhosis cases, suggesting that PSMA overexpression may serve as a highly diagnostic marker for liver tumors.

Furthermore, PSMA PET imaging demonstrated incidental detection of ICC. Kang et al. reported a case of incidental ICC in a 69-year-old man with prostate cancer, where the liver lesion exhibited a notably high PSMA PET SUVmax of 12.8 [7]. Similarly, Sun et al. described an ICC case detected using ¹⁸F-PSMA-1007 PET/MRI, emphasizing the importance of considering alternative diagnoses when unexpected PSMA-avid lesions arise [3]. Another prostate cancer case has also been reported with high PSMA uptake in an incidentally detected ICC that was subsequently treated by SBRT [8].

Despite its diagnostic potential, little research has explored the use of PSMA-targeted radioligand therapy in patients with PSMA PET-positive liver tumors. Hirmas et al. reported two patients with HCC who demonstrated high tumor uptake on ⁶⁸Ga-PSMA-11 PET/CT. However, when treated with a single cycle of 6.9 GBq ¹⁷⁷Lu-PSMA-617, their tumors received only 0.1 and 0.6 Gy of radiation, respectively, indicating subtherapeutic dosing [9]. Unfortunately, as was evident in our patient, it is likely that PSMA expression in the tumor endothelium - rather than directly in the tumor cells - leads to poor retention of PSMA-targeted molecules, resulting in minimal radiation delivery and limited therapeutic efficacy in ICC and other solid tumors [4]. Therefore, for prostate cancer patients with additional PSMA-positive non-prostatic tumors, a multimodal approach is needed.

Multimodal oncologic management

Due to the aggressive nature of ICC, the best treatment outcomes occur if the disease can be detected early and removed surgically, where the five-year recurrence-free survival rate is up to 34% [10]. However, the overwhelming majority of patients present with advanced localized disease that is not amenable to surgery [11]. Systemic treatments, such as chemotherapies, immunotherapies, and targeted therapies, have been minimally effective at extending overall survival [12]. In this case, despite the tumor being potentially resectable, the patient declined surgery, necessitating an alternative definitive treatment approach. The potential of EBRT to provide durable local disease control makes it particularly relevant for patients with unresectable disease or those ineligible for surgical treatment due to personal or medical reasons. A systematic review that analyzed 10 studies on external beam radiation therapy for cholangiocarcinoma found that, among studies focusing solely on ICC, the one-year overall survival rate was 57.1%, while the pooled one-year local control rate was 83.4% [13]. Moreover, the evolution of IMRT, coupled with daily image-guided radiation therapy (IGRT), has significantly improved the therapeutic ratio in ICC by enhancing tumor dose conformity while sparing adjacent normal liver parenchyma. This advancement has mitigated the risk of dose-dependent hepatotoxicity, allowing for higher radiation doses to be delivered safely, thereby improving local control rates and overall survival outcomes in patients with unresectable disease [14].

The decision-making process in this case was guided by a multidisciplinary tumor board and patient preference. Given the solitary nature of the ICC lesion and the patient's concurrent mCRPC, the board favored a localized approach with IMRT (4500 cGy in 15 fractions) rather than initiating systemic chemotherapy, which the patient had also declined after completion of radiotherapy. This decision was influenced by (1) the patient’s explicit refusal of surgery despite its potential curative intent; (2) the favorable response rates of EBRT in non-surgical ICC patients; and (3) the need to preserve systemic treatment options for concurrent mCRPC.

SBRT (35 Gy in five fractions) for pelvic lymph node metastases was recommended as an adjunctive measure to prioritize definitive local therapy. LuPSMA was reintroduced following systemic progression of mCRPC, leading to subsequent PSA decline and tumor burden reduction.

In the present case, a follow-up MRI confirmed a response of ICC to IMRT, with findings corroborated on PSMA PET imaging. This suggests that PSMA PET, which is promising in treatment response assessment for metastatic prostate cancer, could serve as a complementary tool in assessing treatment response in PSMA-avid hepatic tumors [15]. Further research is warranted to validate these findings and establish standardized guidelines for PSMA PET use in this setting.

Emerging evidence indicates that extending Lu-177 PSMA-617 therapy beyond the standard six cycles may offer additional benefits for patients with mCRPC, particularly in achieving optimal tumor radiation doses and prolonged disease control [16,17]. A multicenter study by Seifert et al. demonstrated that patients receiving extended cycles of Lu-177 PSMA-617 experienced a favorable median survival of 31.3 months from the first administration, with preserved efficacy observed upon rechallenge after a treatment break [16]. ​Furthermore, Mader et al. reported that extended radioligand therapy is a feasible option for patients with high-volume residual tumors after completing the standard six cycles, noting improved survival outcomes and an acceptable safety profile [17]. ​These findings strongly support further LuPSMA therapy beyond six cycles for our patient, as extended treatment has been shown to optimize radiation dose delivery, sustain tumor control, and prolong survival. This is particularly relevant given our patient's lymph-node-only disease, where continuous PSMA-targeted radioligand therapy may provide durable therapeutic benefit with minimal toxicity while preserving quality of life. Prior evidence underscores the rationale for prolonged LuPSMA therapy in appropriately selected patients demonstrating ongoing response and good tolerability [18].

## Conclusions

This case underscores the evolving role of PSMA PET beyond prostate cancer staging, demonstrating its ability to detect unexpected malignancies. It also highlights the significance of a multidisciplinary approach integrating theranostics, advanced radiation therapy, and personalized oncology strategies, with post-therapy SPECT/CT playing a crucial role in confirming radioligand uptake and guiding subsequent management decisions.
